# The impact of limited access to electronic medical records on neurosurgical care within the CARICOM countries: A survey and scoping review

**DOI:** 10.1016/j.bas.2023.101747

**Published:** 2023-05-11

**Authors:** Ashia M. Hackett, Christopher O. Adereti, Ariel P. Walker, Ifeanyichukwu Ozobu, Johnnie Petit, Karl R. Waldron, Myron Rolle

**Affiliations:** a Department of Neurosurgery, Barrow Neurological Institute, St. Joseph's Hospital and Medical Center, Phoenix, AZ, USA; b Ross University School of Medicine, Miramar, FL, USA; c Wayne State University School of Medicine, Detroit, MI, USA; d Department of Neurosurgery, Massachusetts General Hospital, Harvard University, Boston, MA, USA; e Rutgers University, Brunswick, NJ, USA

**Keywords:** Global neurosurgery, Electronic medical record, Caribbean community, Low- and middle-income countries, LMIC, Neurosurgical care

## Abstract

**Introduction:**

Global access to electronic medical records (EMRs) continues to grow, however many countries including those within the Caribbean Community (CARICOM) lack access to this system. Minimal research investigating EMR use in this region exists.

**Research question:**

How does limited EMR access impact neurosurgical care within the CARICOM?

**Materials and methods:**

The Cochrane Library, EMBASE, Scopus, PubMed/MEDLINE databases, and grey literature were queried for studies addressing this issue within the CARICOM and low- and/or middle-income countries (LMICs). A comprehensive search for hospitals within the CARICOM was performed and responses to a survey inquiring about neurosurgery availability and EMR access within each facility were recorded.

**Results:**

26 out of 87 surveys were returned leading to a response rate of 29.0%. Among the survey respondents, 57.7% stated neurosurgery was provided at their facility; however, only 38.4% admitted to using an EMR system. Paper charting was the primary means of record keeping for the majority of the facilities (61.5%). The most frequently reported barriers stalling EMR implementation were financial limitations (73.6%) and poor internet access (26.3%). A total of 14 articles were included in the scoping review. Results from these studies suggest that limited EMR access contributes to suboptimal neurosurgical outcomes within the CARICOM and LMICs.

**Discussion and conclusion:**

This paper is the first to address the impact that limited EMR has on neurosurgical outcomes in the CARICOM. The lack of research addressing this issue also highlights the need for ongoing efforts to increase research output focused on EMR accessibility and neurosurgical outcomes in these countries.

## Highlights

• Many countries including those within the Caribbean Community lack access to EMR systems.
• Limited EMR access within the CARICOM has an impact on neurosurgical outcomes.
• There are several benefits to EMR usage, however many barriers also exist.
• This article highlights the need for increased global efforts to address EMR accessibility.

## 1 Introduction

Global neurosurgery is a niche area within global surgery, and in the broader context of global health exists in relative obscurity (Haglund and Fuller, 2019). It is a budding field in which its stakeholders aim to make changes through surgical camps, educational and training programs, health system strengthening projects, health policy changes/development, and advocacy (Haglund and Fuller, 2019). Despite increasing efforts to improve neurosurgical access and quality in unmet regions, many countries–especially low- and/or middle-income countries (LMICs) —continue to face challenges. The lack of neurosurgeons in these countries ascertains that care is not always delivered timely except in instances where patients are prioritized due to their ability to pay for services—leading to further disparities in accessibility on top of availability (Park et al., 2016). Multiple systems-level barriers exist, including poor road infrastructure, limited access to reliable technology, and frequent absence of an electronic medical record (EMR) system for record-keeping (Luan et al., 2021).

EMRs are a systematic collection of patient electronic health information organized to assist the care of patients and groups of patients (Centers for Disease Control and Prevention, 2022). These systems also allow for the management of patients' health information in a form that can be shared across multiple healthcare settings thus improving healthcare quality and serving as an impetus for gold standard development (Centers for Disease Control and Prevention, 2022; Porter et al., 2005; Tweya et al., 2016). EMRs benefit institutions by upholding patients’ health privacy via electronic charts, reducing hospital expenditure, and facilitating clinical research (Cowie et al., 2017; Menachemi and Collum, 2011). Additional core functionalities include results management, order entry and support, and decision support (Blumenthal and Glaser, 2007). Though widely available in hospitals and private practices throughout much of the developed world, many countries including those within the Caribbean Community (CARICOM) still lack access to this electronic system. The CARICOM is a functionally cooperative group of twenty countries: fifteen member states and five associate members all comprising approximately sixteen million citizens from the main ethnic groups of Indigenous Peoples, Africans, Indians, Europeans, Chinese, Portuguese, and Japanese (Kucchal et al., 2020; Rolle et al., 2021; Sastre et al., 2014; CARICOM - Caribbean Community, 2022; Member states and associate members, 2020).

Currently, there remains a dearth of literature addressing the EMR disparity in this region. However, increased access to EMR systems continues to be one of many ongoing efforts to improve health care in this region. Herein, the authors performed a survey and scoping review to assess the impact limited EMR access has on neurosurgical care within the CARICOM while highlighting the need for ongoing research to address this issue.

## 2 Material and methods

### 2.1 Survey

A comprehensive Google search was conducted by two authors (A.H and C.A.) and identified 192 facilities within the 15 CARICOM member countries. The CARICOM nations, including their World Bank economic classifications, are as follows: Low-income: Haiti; Upper middle-income: Belize, Dominica, Grenada, Guyana, Jamaica, Saint Lucia, Suriname, Saint Vincent & the Grenadines; High income: Antigua and Barbuda, The Bahamas, Barbados, and Trinidad and Tobago (Rolle et al., 2021; CARICOM - Caribbean Community, 2022; Member states and associate members, 2020). Hospitals within Trinidad and Tobago (n ​= ​14) were excluded from this study due to a lack of clearance from their research ethics committee. Additional contact information (i.e. office number, email, and website URL) was obtained; duplicate listings (n ​= ​10), facilities without available contact information (n ​= ​72), facilities without personnel that spoke English (n ​= ​6) and facilities that were listed as temporarily/permanently closed (n ​= ​3) were omitted from the study, resulting in 87 facilities. Using Google Forms, authors A.H. and C.A. developed a 16-item survey inquiring about the availability of neurosurgical services and the use of EMR and/or paper charting within each facility. The list of 87 facilities was then divided proportionally between all authors. Hospitals were contacted via email, Skype, Google Meets, Zoom, WhatsApp, or with the use of phone cards (obtained by M.R.). Survey responses were either entered into Google Forms by hospital personnel directly or by authors during phone or video calls with representatives.

### 2.2 Search strategy

Due to the paucity of studies addressing EMR access within the CARICOM, a scoping review was conducted in accordance with the PRISMA-ScR Checklist and methodology framework established by Arksey and O'Malley (Arksey and O'Malley, 2005). The Cochrane Library, Embase, Scopus, PubMed/MEDLINE databases, and grey literature was queried using the search terms: ((CARICOM) OR (Caribbean Community) OR (West Indies) OR (Afro-Caribbean) OR (LMIC) AND (surgery) OR (neurosurgery) OR (neurological surgery) AND (EHR) OR (EMR) OR (electronic health records) OR (electronic medical records)). The search strategy commenced on the 6^th^ of October 2022 and concluded on the 21^st^ of November 2022.

### 2.3 Study selection

**Fig. 1:**
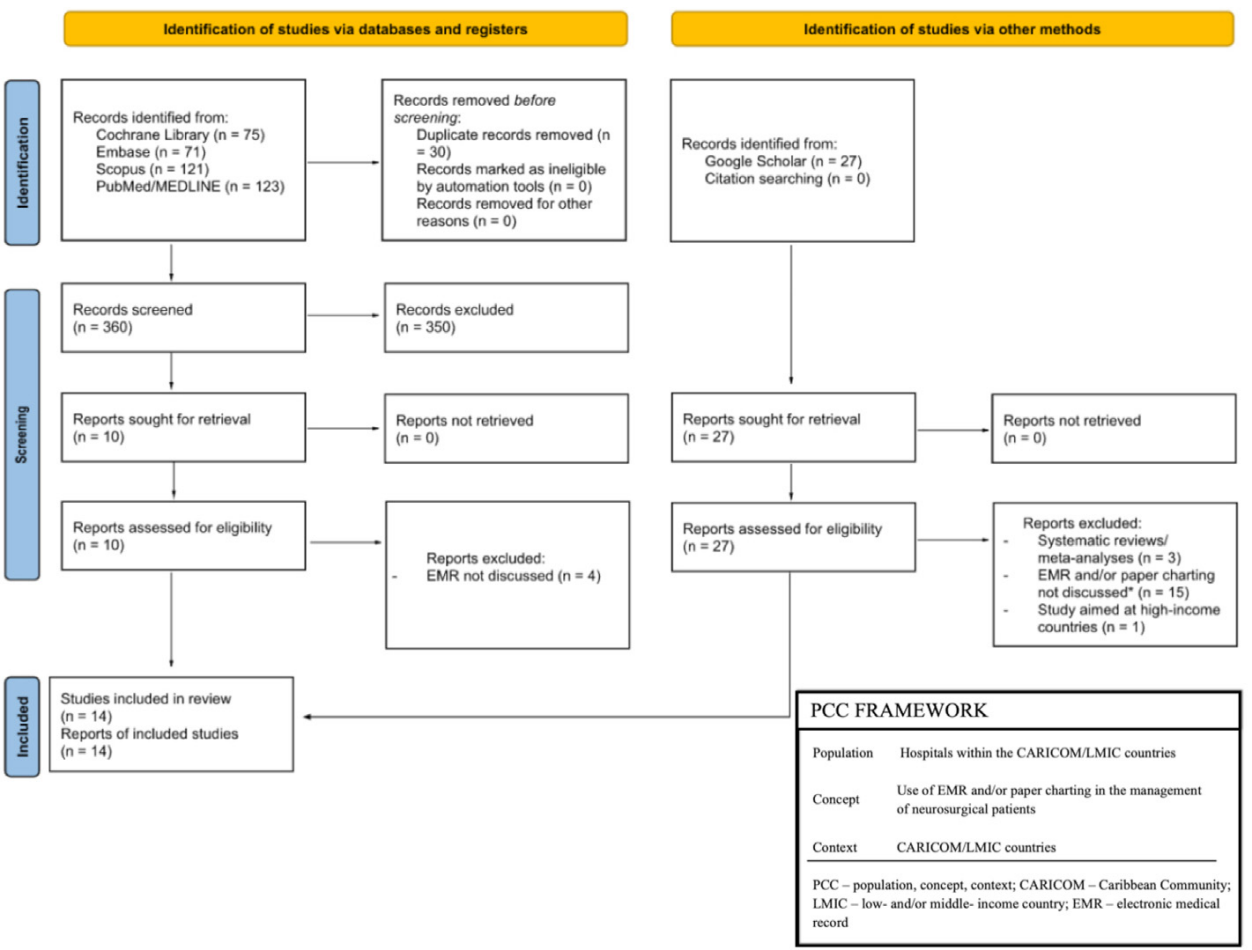
PRISMA flow diagram and PCC framework.

Given the nature of this study, the Population, Concept, and Context (PCC) Framework was used to identify the target population and topic of interest. The inclusion criteria were as follows: (1) Articles addressing neurosurgical and/or surgical access within the CARICOM and LMICs; (2) Articles addressing the use of EMR and/or paper charting in these countries; (3) Non-systematic reviews and/or meta-analyses; (4) Full-text articles written in the English language. Studies not meeting these criteria were excluded. No time period restrictions were applied to the search. The initial search yielded 390 articles. Thereafter, a manual search of Google Scholar and a review of article references were performed to obtain additional studies for article inclusion. Articles identified in the search were imported in Rayyan (https://rayyan.qcri.org/) to allow for screening of studies based on title and abstract in addition to the removal of duplicates, non-full text articles, and non-English articles. Two authors (A.H. and C.A.) independently reviewed potentially eligible titles and abstracts and contributed to the review process. Disagreements between the reviewers were infrequent and resolved through discussions. A visual of the search strategy is displayed in Fig. 1.

## 3 Results

### 3.1 Survey responses

In total 26 (16 complete and 10 incomplete) out of 87 surveys were returned leading to a response rate of 29.0%. Responses were obtained from Haiti (n ​= ​9), Grenada (n ​= ​2), Dominica (n ​= ​1), Guyana (n ​= ​3), Bahamas (n ​= ​3), Montserrat (n ​= ​1), Jamaica (n ​= ​2), Belize (n ​= ​2), Saint Kitts & Nevis (n ​= ​2) and Antigua & Barbuda (n ​= ​1) (Table 1). Results showed that 57.7% (n ​= ​15) of hospitals provide direct neurosurgical care at their facility, whereas 6 out of the 11 that do not offer neurosurgical services typically refer their patients to hospitals that do. EMR systems are used by 38.4% (n ​= ​10) of the facilities, 4 of which report using both EMR and paper charts. However, 57.6% of respondents (n ​= ​15) rely solely on paper charts. Of the 26 hospitals that responded to the survey, 12 were in rural communities, 11 in urban communities, and 3 did not report it. (Table 1). When sending or receiving records, most facilities use more than one method; however 92.3% of respondents admit to sending and receiving hard copy referrals to and from outside facilities. Other methods for sharing records include: phone calls (n ​= ​2), emails (n ​= ​4), fax (n ​= ​1), and WhatsApp (n ​= ​1). Picture archiving and communication systems (PACS) (n ​= ​9) and hard copy (n ​= ​5) are the main modalities used to view labs and images; other modalities that are used include phone (n ​= ​2), CD (n ​= ​1), and EMR (n ​= ​1) (Table 2). The average duration to receive imaging or lab results is less than or equal to 1 ​h for 30.7% (n ​= ​8) of the facilities, 3–6 ​h for 11.5% (n ​= ​3), and up to 24 ​h for 0.07% (n ​= ​2); 2 facilities said the timing was dependent on the type of lab or imaging ordered and 11 hospitals did not provide a response. Only 1 facility reported that image results are linked to their EMR system. Among the 10 facilities that use EMR systems, 40.0% (n ​= ​4) reported that physicians directly chart into the EMR system, 30.0% (n ​= ​3) reported that paper charts are used initially and later transferred into the EMR system, 10% (n ​= ​1) reported that direct input of patient information into the EMR system varies depending on the physician. The remaining 2 hospitals failed to provide a response. Additionally, 8 of these 10 facilities reported having a computer in the hospital which is located in an office (n ​= ​5), operating room (n ​= ​1), patient room (n ​= ​2), or nursing station (n ​= ​2). No response on computer access was provided by 2 hospitals. Input delays due to a lack of computer access, internet access, or electricity were reported by 40.0% (n ​= ​4) of respondents, whereas 2 facilities (20.0%) reported never or almost never having input delays. 4 facilities did not provide a response to input delays. 5 out of the 10 facilities that use EMR systems reported having a preference for EMRs over paper charting, 2 reported no reference, and 3 did not provide a response. Among the remaining 16 hospitals that do not use EMR systems, 5 admitted to considering switching to EMR, 2 have not considered it, and 9 failed to answer. Hospitals that use both EMR systems and paper charting or only use paper charting reported barriers to EMR implementation. Reasons provided were: financial limitations (n ​= ​14) inadequate internet access (n ​= ​5), limited electricity (n ​= ​2), lack of hardware infrastructure (n ​= ​2), inadequate training (n ​= ​3), and/or stakeholder acceptance (n ​= ​2) (Table 3). Among the 19 hospitals that use either EMR or paper charting or paper charting solely, 8 reported having reservations about transitioning to EMR full time. These reservations vary between facilities and include confidentiality concerns, affordability, unreliable information technology platforms, technical team availability when issues arise, and a need for a legislative framework.

**Table 1:** Survey results by country that offer neurosurgical care and current EMR status.

Country Name (no. of surveys submitted)	Neurosurgical Care		EMR Usage				Community Type		
	Yes (n)	No (n)	Yes (n)	No (n)	Both (n)	NR (n)	Urban (n)	Rural (n)	NR (n)
Antigua & Barbuda (n ​= ​1)	1	0	0	1	0	0	0	1	0
Bahamas (n ​= ​3)	3	0	0	3	0	0	3	0	0
Belize (n ​= ​2)	2	0	1	1	0	0	2	0	0
Dominica (n ​= ​1)	0	1	0	1	0	0	0	1	0
Grenada (n ​= ​2)	1	1	0	1	0	1	1	1	0
Guyana (n ​= ​3)	1	2	0	3	0	0	0	2	1
Haiti (n ​= ​9)	4	5	3	2	4	0	4	4	1
Jamaica (n ​= ​2)	2	0	2	0	0	0	1	1	0
Montserrat (n ​= ​1)	0	1	0	1	0	0	0	1	0
Saint Kitts & Nevis (n ​= ​2)	1	1	0	2	0	0	0	1	1
**Total Surveys: 26**	**15**	**11**	**6**	**15**	**4**	**1**	**11**	**12**	**3**

Abbreviations: NR – Not reported; EMR – Electronic medical record.

**Table 2:** Types of modalities used by facilities for medical record sharing and viewing labs and images.

Modality Type	Medical Record Sharing Frequency (%)
Hard Copy	24 (92.3%)
Email	4 (15.3%)
Phone Calls	2 (7.6%)
Fax	1 (3.8%)
WhatsApp	1 (3.8%)
Not Reported	1 (3.8%)
	**Imaging and Lab Viewing** **Frequency (%)**
PACS	9 (34.6%)
Hard Copy	5 (19.2%)
Phone	2 (7.6%)
CD	1 (3.8%)
EMR	1 (3.8%)
Not Reported	9 (34.6%)

**Table 3:** Barriers to EMR implementation reported by hospitals that still use paper charts.

Barrier Type	Reported Frequency (%)
Financial	14 (73.6%)
Inadequate Internet Access	5 (26.3%)
Limited Electricity	2 (10.5%)
Inadequate Hardware Infrastructure	2 (10.5%)
Inadequate Training	3 (15.7%)
Stakeholder Acceptance	2 (10.5%)
Not Reported	5 (26.3%)

### 3.2 Literature search

We identified 390 articles using the Cochrane Library, Embase, Scopus, and PubMed/MEDLINE databases. 30 duplicates were identified and excluded. During the screening by abstract and title phase, 350 records failed to meet the inclusion criteria and were also excluded. After full-text article screening (n ​= ​10), 4 additional reports were excluded and the remaining 6 were then included in the scoping review. A similar process was performed using Google Scholar. A manual search identified 27 records and each was assessed for eligibility. 19 records failing to meet the inclusion criteria were excluded and included the remaining 8 articles in this scoping review. No additional records were obtained via a citation search of included articles.

### 3.3 Scoping review

A total of 14 articles were included in this scoping review (Luan et al., 2021; Shapiro and Kamal, 2022; Ferry et al., 2021; Dasari et al., 2016a, 2016b; Grant et al., 2021; Yancey et al., 2019; Mukherjee et al., 2022; Williams and Boren, 2008; Gyedu et al., 2015; Spence et al., 2019; Meara et al., 2015; Rock et al., 2020; Quinsey et al., 2018) (Table 4). Three overarching themes were identified: (1) the overall benefit of EMR usage is substantial; (2) extreme barriers to implementing EMR systems in LMICs exist; and (3) there is a direct correlation between research efforts and EMR usage. The most common theme discussed among the articles (n ​= ​9) was the benefit of using an EMR system. Department efficiency, reduced long-term financial cost, and enhanced data collection by switching from paper charting to electronic charting are underscored throughout these articles. 7 articles highlighted barriers to EMR implementation, such as funding, stakeholder acceptance, and technical challenges. The relationship between research and EMR usage was analyzed in 4 of the articles, in which they identified the difficulty of conducting research in environments that do not use EMR systems and the effect this can have on health disparities.

**Table 4:** Scoping review articles and results.

Author (Year)	Journal	Geographic Subgroup(s) Included in Study	Benefits of EMR	Barriers to EMR Implementation	Role of EMR in Research
Dasari et al. (2016)	World J World Journal of Surgery	Latin America	Increased efficiency and accuracy of patient data recording, including more timely completion of the patient chart.	Concerns amongst all stakeholders regarding reinforcement, roles, and responsibilities for using a novel EHR tool, and technology infrastructure.	Ability to conduct retrospective research
Dasari et al. (2016)	Annals of Global Health	Latin America	Not discussed.	Skepticism regarding aspects of EMR, thus requiring extensive pre-implementation meetings, focus groups, and discussions to address the feasibility and acceptance of implementing this tool into the hospital. High cost of software support, specifically for troubleshooting and customization needed in each country.	Not discussed.
Ferry et al. (2021)	Plastic and Reconstructive Surgery – Global Open	Central and South Asia East and Southeast Asia Europe and North America Latin America and the Caribbean North Africa and West Asia Sub-Saharan Africa	Increased speed of documentation, easier access to charts, and easier transfer of charts across care teams (not referrals). Enhanced data reliability and ability to double-check data input.	Financial constraints; lack of tech-savvy staff and high clinical volume leads to decreased workflow.	Not discussed.
Grant et al. (2021)	Injury	Sub-Saharan Africa	The electronic system provides an opportunity to improve care for all patients by reducing duplication of patient records and improving accessibility of patient records by providing reliable means of linking records from repeated visits. Increases documentation time and efficiency.	Not discussed.	Not discussed.
Gyedu et al. (2015)	International Journal of Surgery	Sub-Saharan Africa	Long-term staff efficiency, referral efficiency, and cost savings.	Not discussed.	Not discussed.
Luan et al. (2021)	International Journal of Surgery	Not specified	Not discussed.	Limited access to reliable technology and insufficient funds to invest in resources,	Not discussed.
Meara et al. (2015)	The Lancet Commissions	Not specified	Not discussed.	Lack of power or electricity; limited access to computers or the internet.	Improves clinical outcomes; enhances both data collection and process monitoring.
Mukherjee et al. (2022)	Child's Nervous System	South Asia	Flagged records to assist in data collection, correction, or verification Long term financial cost decreased Allowing better assessment and ability to identify areas that need improvement	Competing demands on neurosurgeon's time Local technical support; cost of system maintenance; increased cost of EMR with greater provider utilization High start-up cost and design cost Corrupted or locked records; incorrect or incomplete data entry	Enhanced analytic and research capabilities.
Quinsey et al. (2018)	Neurosurgical Focus	Sub-Saharan Africa	Not discussed.	Not discussed.	Absence of EMRs and poor/absent documentation within paper charting stalls many research efforts. Without consistent documentation, retrospective data collection becomes futile and necessitates real-time data collection personnel; the absence of reliable medical record documentation in many LMICs can make retrospective studies unrealistic.
Rock et al. (2020)	World Neurosurgery	Southeast Asia	Not discussed	Not discussed	Not discussed
Shapiro and Kamal (2022)	The Journal of Hand Surgery	Not specified	Provides access to critical patient information for clinical care or providing surgeons with information for staged procedures and as a “sign out” for future surgeons; improve patient safety and quality of care delivery, with an enhanced ability to review perioperative complications, improve preoperative optimization and resource use, and derive insights that inform future outreach efforts Provides the structure and process to improve data collection and tracking rates.	Not discussed	Not discussed
Spence et al. (2019)	The Lancet Oncology	The Caribbean	Promotes reliable health information exchange systems to better coordinate care and prevention throughout the entire Caribbean region.	Not discussed	Not discussed
Williams and Boren (2008)	International Journal of Information Management	Sub-Saharan Africa	Easy monitoring/treatment of diseases; reduces or prevents the possibility of mixing patient's records and ensures confidentiality and privacy of documents. Easy accessibility and retrieval of medical documents; storage, communication, and consistency in work performance.	Hesitancy amongst stakeholders— simply not prepared to adopt an EMR system and waiting to see the importance of EMR implementation. Insufficient cost in terms of training personnel, maintenance, and cost of acquiring the EMR equipment. Lack of power or electricity, limited knowledge of technology and equipment.	Not discussed.
Yancey et al. (2019)	International Journal of Pediatric Otorhinolaryngology	Sub-Saharan Africa The Caribbean	The use of EMR supports patient follow-up and allows for remote consultation.	Not discussed.	Not discussed.

## 4 Discussion

This scoping review highlights three points: the benefits of utilizing an EMR system, the barriers to widespread implementation of this tool, and the role EMR plays within research. Currently, research focused on the use of EMR within the CARICOM is scarce. However, the studies included in our review collectively identify the barriers often encountered in hospitals within most LMICs. As such, each point is important to consider when addressing the impact of EMR shortages within the CARICOM.

### 4.1 Benefits of EMR use

Most of the articles included in this review addressed the benefits of EMR use. However, none of the articles described these benefits in any of the facilities included in our survey. Despite this, two frequently reported benefits of EMR systems in these studies were departmental efficiency and enhanced patient documentation. Using electronic systems can allow for the timely completion of patients' charting via the use of drop-down menus and templates (Shapiro and Kamal, 2022; Ferry et al., 2021; Dasari et al., 2016a). In a study conducted by Grant et al., the authors performed a retrospective chart review of trauma patients at Mbarara Regional Referral Hospital in Uganda. They found that there was an increased incidence rate ratio of 20.9 (95% CI 15.7–27.6, p ​< ​0.001) in completed trauma patient documentation following the implementation of a trauma registry and electronic patient registration system compared to the use of paper charting (Grant et al., 2021). Once clinical providers have learned to use EMRs effectively, it can streamline access to critical information in a patient's charts which guides clinical decision-making (Shapiro and Kamal, 2022; Grant et al., 2021). The ability to share patients' charts across care teams within a single facility can also lead to more coherent medical practice (Ferry et al., 2021). Furthermore, the implementation of security safeguards within EMR systems helps to ensure that patients' medical records are protected during exchanges between medical personnel (Kruse et al., 2017).

Aside from the security provided through EMRs, this system offers plenty of organizational benefits. Many local surgeons rely on inconsistent paper documentation to maintain patients' contact information and schedules, potentially contributing to the loss of follow-up (Luan et al., 2021). However, EMR systems have been shown to support patient follow-up and allow for remote consultations (Yancey et al., 2019). In a study describing the design, implementation, and adoption of an EMR system by the pediatric neurosurgical department at the National Institute of Neurosciences and Hospital in Dhaka, Bangladesh, patient tracking, hospital discharge, and outpatient follow-up were easily managed after EMR implementation (Mukherjee et al., 2022). This can be attributed to easier monitoring of a patient's record; when there is only one chart in an EMR system it can link records from repeated visits and maintain all patient information in one location (Grant et al., 2021; Williams and Boren, 2008). Ensuring better patient documentation and more consistent follow-up can lead to improved patient clinical outcomes.

Due to the ratio disparity among providers-to-patients commonly present in LMICs, the volume of surgical referrals in these regions is often more prominent. Gyedu et al. specifically assessed the quality of referrals for surgery to Komfo Anokye Teaching Hospital in Kumasi, Ghana, and highlighted the importance of the quality of the information provided in referral forms and completeness (Gyedu et al., 2015). Compliance with referral forms and immediate processing can be ensured when referral systems are built into EMRs, as demonstrated in high-income countries, leading to reduced waiting times and improved staff efficiency (Gyedu et al., 2015). In a study by Rolle et al., St. Kitts and Montserrat were noted to lack full-time neurosurgeons on either island (Rolle et al., 2021). Patients living in one of these countries requiring neurosurgical care may be referred to a hospital on a separate island. Establishing compatible EMR systems throughout the entire Caribbean region would promote reliable health information exchange systems between islands to ultimately better coordinate care (Spence et al., 2019).

### 4.2 Barriers to EMR implementation

Notwithstanding the benefits of EMR use, many barriers to implementing this system in LMICs exist. Among the studies included in the review, the most common barriers reported were insufficient funding and lack of infrastructure to support EMR use. Adequate funding is needed for hardware, EMR software, implementation assistance, training, and ongoing network fees and maintenance (How much is this going, 2014). Though funding may present initially as a barrier to EMR implementation, through data-based quality improvement initiatives that save time and money and improve patient care and outcomes, Mukherjee and company point out there is a long-term cost benefit to using EMR(Mukherjee et al., 2022). Irrespective of the financial costs, the lack of infrastructure to sustain EMR systems makes implementation challenging. Reliable electricity, a necessity for any healthcare system, has an even higher demand in the setting of EMR utilization (Williams and Boren, 2008; Meara et al., 2015). In one survey completed at a teaching hospital in Accra, Ghana, the researchers reported that limited electricity was the greatest challenge stalling EMR implementation (Williams and Boren, 2008). They further noted that most countries in sub-Saharan Africa and other poor nations lack the experts, funds, and infrastructure necessary for the widespread implementation to ensure continuity of care (Williams and Boren, 2008). Similarly in the present review of LMICs, limited access to the internet, technology-savvy personnel, software training, and working computers and/or laptops is a barrier faced by many CARICOM countries (Luan et al., 2021; Ferry et al., 2021; Meara et al., 2015). Another study by Dasari et al. found that there was skepticism among clinical providers and hospital administrators regarding aspects of EMR, thus requiring extensive pre-implementation meetings, focus groups, and discussions to address the feasibility and acceptance of implementing this tool in the hospital (Dasari et al., 2016b). Hesitancy among stakeholders was also noted in other articles, pointing out the competing demands on neurosurgeons’ time to use a new system and simply not being prepared to adapt to this system (Mukherjee et al., 2022; Williams and Boren, 2008).

Through government initiatives and international collaborations, many of these barriers can be overcome (Shapiro and Kamal, 2022; Williams and Boren, 2008). Global health partnerships, such as “twinning” is a way for neurosurgery departments in specific countries to facilitate an exchange of information, resources, and research which can enhance EMR implementation in LMICs (Mukherjee et al., 2022). As previously demonstrated in the CARICOM, pan-Caribbean partnerships have proven to be successful ways of overcoming health barriers (Theodore-Gandi and Barclay, 2008; Hospedales et al., 2011). Elimination of indigenous polio, measles, and rubella, its response to HIV/AIDS, and more recently, its response to the COVID-19 pandemic, each show how important collaborative efforts are for this region (CARICOM secretariat strengthening Focus On Partnerships, 2022; Chattu and Knight, 2019). By means of well-organized collaborations, EMR systems can be successfully implemented and ultimately improve the quality of neurosurgical care in the CARICOM nations.

### 4.3 The role of EMR systems in research

In addition to the health information security provided through EMR, the availability of this system allows for increased research opportunities that can, in turn, address health issues at both the individual and population levels while facilitating access to research funding (Meara et al., 2015). Greater research capabilities in turn facilitate physicians' understanding of various clinical pathologies which improves their ability to treat patients (Mukherjee et al., 2022). Conversely, the absence of EMR systems and the reliance on paper charting makes data acquisition and analysis more challenging and hinders retrospective data collection thus necessitating real-time data collection personnel (Rock et al., 2020; Quinsey et al., 2018). In a study by Whiffin et al., the authors described neurosurgeons' experience with conducting and disseminating clinical research in LMICs (Whiffin et al., 2021). They noted that limited EMR access impedes research efforts in these countries and makes retrieval of relevant clinical material challenging (Whiffin et al., 2021).

### 4.4 EMRs within the CARICOM

The results from the present survey give an overall picture of the state of EMR use within the CARICOM countries. While the benefits of EMR are plenty, many countries do not use this system. The barriers identified in our survey are similarly reported studies focused on other LMICs. These barriers include but are not limited to, a lack of the infrastructure to support EMRs, funding, and stakeholder acceptance. Though some countries within the CARICOM use EMR systems, when referring patients, records cannot be transferred electronically due to the lack of EMR systems across many hospitals. This may result in a delay of care and miscommunication. PACS were the most commonly used imaging software, however, some countries still rely on hard copies. St. George Hospital in Grenada reported receiving imaging results via phone. Neurosurgical planning and clinical decision-making are heavily reliant on radiological imaging, thus improving the way clinical providers view imaging can be extremely beneficial (Wang et al., 2015; Duncan et al., 2016).

In an effort to assess how barriers to EMR access affect neurosurgical outcomes in the CARICOM, survey methodology was used to draw conclusions. Despite a limited response rate, results suggest that the implementation of an EMR system would be beneficial to neurosurgical practice and improve outcomes therein. Though not studied directly, these findings likely translate to other specialties as well. The benefits of EMR implementation are multifold. In a previous study addressing timely access to neurosurgical care in the Caribbean using geospatial analysis, the authors identified only 16 hospitals in this region that provided neurosurgical care (Rolle et al., 2021). Results from this study suggest that geographical barriers may limit equitable access to neurosurgical care in the Caribbean (Rolle et al., 2021). These findings further stress the significance of implementing an EMR system as access to EMR would allow for effective communication and increased continuity of care amongst all patients, especially those facing geospatial barriers. The implementation and sustainment of an EMR system in health facilities would bolster clinical workflow and patient outcomes as implied in the survey responses.

Few CARICOM Nations including Jamaica, The Bahamas, and Trinidad and Tobago have made plans to implement an EMR system nationwide, however, the time to implementation of this system remains unclear (Spence et al., 2019). In Barbados, electronic health records are used in primary care but are not yet available in public hospitals (Spence et al., 2019). The authors conclude that both horizontal collaborations among island nations in the region and vertical collaboration between small island nations and academic and policy collaborators from developed countries will be important contributions to optimizing resources in the Caribbean (Spence et al., 2019).

### 4.5 Limitations

This study includes several limitations. First, the search strategy included only articles written in the English language. This was done to avoid any possibility of misinformation in text translation. However, this may have limited the number of otherwise eligible studies included in the scoping review. Second, due to limited research addressing medical care and EMR access in the CARICOM, the current literature search was expanded to include LMICs. This, therefore, limits the internal validity of the present findings as it applies to neurosurgical care in the CARICOM region specifically. Despite the inclusion of articles addressing EMR barriers outside of the CARICOM, the number of studies meeting the inclusion criteria was small. Third, a manual Google search for all hospitals in the CARICOM was conducted by two authors and thus an erroneous omission of some hospitals may have occurred. As such, the complete count of hospitals identified by the authors may not include all facilities where neurosurgical care is provided within the CARICOM. Fourth, the response rate to the present questionnaire was limited, despite multiple hospital reminders to complete the survey. This low response rate does not adequately reflect the barriers to EMR access or their effects on neurosurgical outcomes encountered in other hospitals and countries within the CARICOM; therefore, the generalizability of the present findings is greatly reduced. Further studies which include a higher response rate can add to the present findings. Lastly, due to the fee associated with international phone calls, calling cards and virtual platforms were used to speak directly with hospital representatives. However, challenges such as limited call minutes and suboptimal internet connection were encountered and occasionally interfered with communication with hospital representatives.

### 4.6 Conclusion

Limited access to EMR systems highlights an unmet need in the CARICOM countries. This study underscores the benefits of EMR usage, barriers to EMR access, and difficulties conducting research faced by hospitals within this region and highlights the need for increased global efforts to address EMR accessibility and neurosurgical care in these countries. To the authors’ knowledge, this is the first study that specifically aimed to identify barriers to EMR access in the CARICOM. The benefits of EMR systems in medical practice have been recognized globally. Implementing this system in the CARICOM requires familiarity with the system, adequate healthcare funding, good planning and management, sustainable internet access, and coordinated efforts involving all stakeholders.

## Declaration of competing interest

The authors have no personal, financial, or institutional interest in any of the drugs, materials, or devices described in this manuscript.
